# Post Cholecystectomy Choledochoduodenal Fistula: A Case Report

**DOI:** 10.31729/jnma.8815

**Published:** 2024-11-30

**Authors:** Anip Joshi, Samita Shakya, Surakshya Thapa, Rifka Sultan, Alish Rajbhandari, Piya Baral

**Affiliations:** 1 Department of Surgery, Bir Hospital, National Academy of Medical Sciences, Mahabaoudha, Kathmandu, Nepal; 2 Bir nospiTal, NaTional Academy of Medical Sciences, Mahabaoudha, Kathmandu, Nepal; 3 Ludwig Maximilian University, Geschwister-Scholl-Platz 1, Munich, Germany

**Keywords:** *choledochoduodenal fistula*, *choledocholithiasis*, *CBD exploration*

## Abstract

Choledochoduodenal fistula is an abnormal connection between the common bile duct and the duodenum. The commonest cause is cholecystolithiasis, however, other causes are iatrogenic factors, bile duct stones (choledocholithiasis), and chronic duodenal ulcers. Here, we report a case of choledochoduodenal fistula secondary to long standing choledocholithiasis post cholecystectomy who presented with intermittent abdominal pain in the past three years which revealed choledochoduodenal fistula during Endoscopic Retrograde Cholangiopancreatography. As the patient had recurrent pain along with choledocholithiasis, surgical intervention was indicated. Choledochoduodenal fistula is suspected in case of recurrent cholangitis and surgery is recommended for refractory and complicated cases. Surgical treatment is also recommended for larger fistulas and especially with non-resolving medical treatment. This case highlights the treatment option for choledocholithiasis with choledochoduodenal fistula.

## INTRODUCTION

Choledochoduodenal fistulas (CDFs) occur when the common bile duct (CBD) and the duodenum form an abnormal connection. CDF accounts for only 8.6% of enterobiliary fistulas.^1^ The majority of cases, nearly 90%, are caused by cholecystolithiasis,although additional etiologies include iatrogenic factors, bile duct stones and chronic duodenal ulcers.^2^ CDF primarily affects older individuals and frequently presents with non-specific symptoms such as abdominal pain, vomiting, and jaundice which can complicate timely diagnosis.^3^ Patients with symptoms or those at risk of complications such as cholangitis, sepsis, or biliary obstruction usually require surgical intervention. We present a case of a 74-year-old male post cholecystectomy who presented with choledochoduodenal fistula.

## CASE REPORT

**Figure 1:**
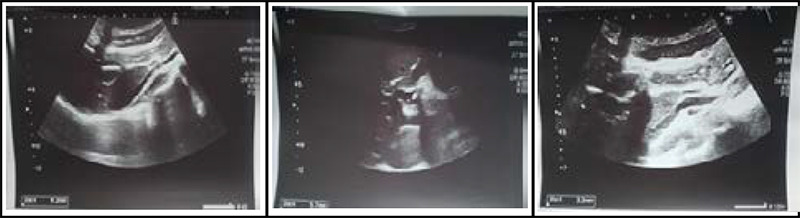
Abdominal USG showing a dilated CBD and choledocholithiasis

**Figure 2:**
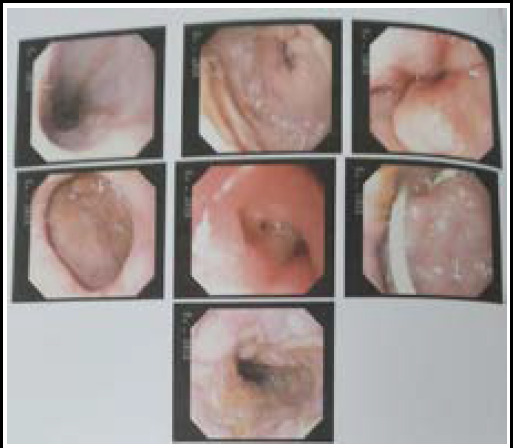
ERCP showing reflex oesophagitis LAC, stented CBD and choledocholithiasis with choledochoduodenal fistula

**Figure 3:**
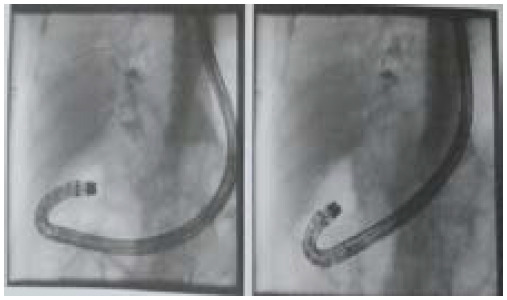
ERCP showing reflex oesophagitis LAC, stented CBD and choledocholithiasis with choledochoduodenal fistula

In this report, we present a case of a 74-year-old male with no known chronic comorbidities with on and off epigastric pain for three years. He had a previous history of open cholecystectomy 10 years back. On clinical examination, he was hemodynamically stable, and the general physical condition was normal with a right subcostal healthy scar. Laboratory investigations showed lower WBC count (4570/Cu.mm.), total bilirubin (1.6mg/dl), SGOT (47IU/L), alkaline phosphatase (525), and amylase within normal limits. USG (Figure 1) revealed a 10 mm dilated CBD with a stent in situ and choledocholithiasis with a few calculi, the largest measuring 6 mm. Endoscopic Retrograde Cholangiopancreatography (ERCP) (Figure 2 and 3) showed a dilated CBD with a 7Fr 5cm plastic stent through fistulous opening at the junction of first and second parts of duodenum with diagnosis of choledocholithiasis with choledochoduodenal fistula and reflux oesophagitis LA-C. CT scan revealed choledocholithiasis with features of biliary obstruction proximally, pneumobilia and dilated main pancreatic duct (MPD).

**Figure 4:**
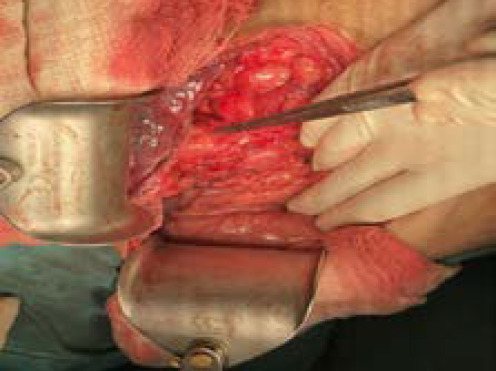
Demonstration of dilated Common bile duct

**Figure 5:**
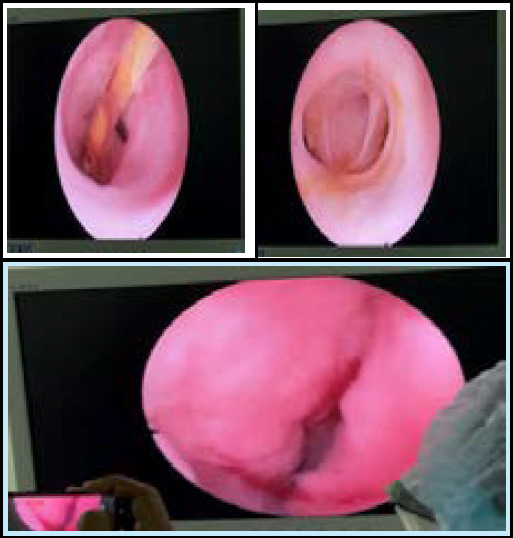
Choledochoscopy (A) stent visualisation (B) proximal hepatic ducts (C) opening into duodenum

He underwent exploration of the common bile duct, intra-operative choledochoscopy, and placement of a T-tube drainage. The operative findings revealed a dilated 2cm common bile duct (Figure 4) with stent in situ, a choledochoduodenal fistula connecting first part of duodenum with the distal CBD and two stones in the duodenum, each measuring 0.3×0.3 cm^2^. A choledochoscope was introducedrevealing a stent in situ (Figure 5a) which was removed and visualising the proximal and distal common bile duct for stone clearance confirmation (Figure 5b and 5c).

The choledochoduodenal fistula was traced distally through CBD upto the duodenum (Figure 5c). The common bile duct was cleared of the stones and choledochotomy closure was done over T-tube.

The T-tube cholangiogram was done on post operative day 10 which did not reveal any retained stones or other tracts. The T-tube was removed on post operative day 14. The post operative course of the patient was uneventful and the patient was discharged.

## DISCUSSION

Choledochoduodenal fistula is an abnormal pathway connecting the CBD with the duodenum. It is a rare complication of long standing choledocholithiasis. As a result of this rare condition, bile bypasses the normal drainage pathway and flows directly into the intestinal tract. Most patients present with a history of jaundice, abdominal pain and fever, and the fistula is found incidentally during endoscopy.^4^ The most common causes of a choledochoduodenal fistula are cancer, trauma, amoebic infections, and peptic ulcers.^5^ Our patient supposedly developed the fistula as a result of longstanding choledocholithiasis. Imaging methods such as computed tomography (CT) are essential in diagnosing CDF, revealing the abnormal communication between the CBD and the duodenum. Other diagnostic techniques, including magnetic resonance cholangiopancreatography (MRCP) and endoscopic retrograde cholangiopancreatography (ERCP), are also useful in evaluating the anatomy and extent of the fistula.

The management of CDF is not standardised as it varies with size, etiology and other factors. It is reasonable to attempt medical treatment of smaller fistulas among those less than 0.5 cm. Surgical treatment is recommended for larger fistulas and especially with non-resolving medical treatment.^4^ Non-specific symptoms such as abdominal pain, vomiting, and jaundice makes the diagnosis very difficult. Pneumobilia seen on CT scans is crucial in the diagnosis.^5^ In our case, as the ERCP failed for stone extraction and the patient had recurrent cholangitis, he was advised for surgical intervention.

Diagnostics and treatment will pose some challenges when CDF is associated with bleeding or gastric outlet obstruction in patients who have unstable hemodynamics. Bleeding due to erosion of the gastroduodenal artery is the most prevalent complication among patients of peptic ulcer disease, while gastric outlet obstruction may develop as a consequence of inflammatory processes. Emergency endoscopy is typically the first line treatment for bleeding, but if pyloric stenosis obstructs visualisation, contrast-enhanced CT angiography is preferred to locate and manage the hemorrhage.^6^ The surgery is recommended for for larger fistulas and especially with non-resolving medical treatment and in complicated cases as failed ERCP for stone extraction in the cases associated with choledocholithiasis.
